# Trajectories of HbA1c Levels in Children and Youth with Type 1 Diabetes

**DOI:** 10.1371/journal.pone.0109109

**Published:** 2014-10-02

**Authors:** Orit Pinhas-Hamiel, Uri Hamiel, Valentina Boyko, Chana Graph-Barel, Brian Reichman, Liat Lerner-Geva

**Affiliations:** 1 Maccabi Juvenile Diabetes Center, Raanana, Israel; 2 Pediatric Endocrine and Diabetes Unit, Edmond and Lily Safra Children's Hospital, Sheba Medical Center, Tel Hashomer, Israel; 3 Sackler School of Medicine, Tel-Aviv University, Tel Aviv, Israel; 4 The Women and Children's Health Research Unit, Gertner Institute, Tel Hashomer, Israel; San Raffaele Hospital, Italy

## Abstract

**Purpose:**

To illustrate the distribution of Hemoglobin A1c (HbA1c) levels according to age and gender among children, adolescents and youth with type 1 diabetes (T1DM).

**Methods:**

Consecutive HbA1c measurements of 349 patients, aged 2 to 30 years with T1DM were obtained from 1995 through 2010. Measurement from patients diagnosed with celiac disease (n = 20), eating disorders (n = 41) and hemoglobinopathy (n = 1) were excluded. The study sample comprised 4815 measurements of HbA1c from 287 patients. Regression percentiles of HbA1c were calculated as a function of age and gender by the quantile regression method using the SAS procedure QUANTREG.

**Results:**

Crude percentiles of HbA1c as a function of age and gender, and the modeled curves produced using quantile regression showed good concordance. The curves show a decline in HbA1c levels from age 2 to 4 years at each percentile. Thereafter, there is a gradual increase during the prepubertal years with a peak at ages 12 to 14 years. HbA1c levels subsequently decline to the lowest values in the third decade. Curves of females and males followed closely, with females having HbA1c levels about 0.1% (1.1 mmol/mol) higher in the 25^th^ 50^th^ and 75^th^ percentiles.

**Conclusion:**

We constructed age-specific distribution curves for HbA1c levels for patients with T1DM. These percentiles may be used to demonstrate the individual patient's measurements longitudinally compared with age-matched patients.

## Introduction

Type 1 diabetes (T1DM) is a lifelong disease affecting children and adolescents that ultimately can lead to significant morbidity. Measuring the hemoglobin A1c (HbA1c) is the standard of care for assessing long-term glycemic control of patients with diabetes, describing the average glycemic level. Lowering HbA1c levels to below or around 7% (53 mmol/mol) has been shown to reduce microvascular and macrovascular complications of diabetes [1], thus HbA1c measurement also provides a treatment goal. According to the guidelines of the International Society of Pediatrics and Adolescents (ISPAD) treatment of T1DM should target HbA1c level of <7.5% (<58 mmol/mol), if achieved without severe episodes of hypoglycemia [2]. The American Diabetes Association (ADA) recommend a target values for HbA1c in relation to age as follows: HbA1c<8.5% (<69 mmol/mol) at age <6 years, <8 (<64 mmol/mol) % at 6 to 12 years, <7.5% (<58 mmol/mol) at 13 to 18 years and <7% (<53 mmol/mol) in adults [3].

Nevertheless, current studies show that only minority of the subjects achieve the goals of ISPAD [4]. HbA1c levels above 9.5% (<80 mmol/mol), reflecting poor glycemic control, were found in 17% of youth in the SEARCH for diabetes in Youth Study conducted in the United States among 3,947 individuals with T1DM [5]. The average HbA1c in the Hvidore study of 2,873 children from 18 countries in Europe was 8.6 (70 mmol/mol) [6]. Results from the Type 1 Diabetes Exchange Clinic Network, including 13,316 participants revealed that overall, only 32% met the ADA targets for their age group and 25% met the ISPAD goals. The proportions meeting the ADA goals were 64% for the under-6-year age group, 43% for 6- to 12-year-olds, and 21% for the 13- to 19-year-olds [7].

Despite recent advances in diabetes therapy including the new insulin analogs, insulin intensification strategies and newest therapeutic technologies such as continuous glucose monitoring system (CGMS) many patients fail to reach or maintain target HbA1c [8]. Persistent poor glycemic control has been documented also in adults with T1DM [9]. During a 4 year follow-up, 30% of subjects from the selected cohort of the Diabetes Control and Complications trial (DCCT) had average HbA1c levels above 8.5% (<69 mmol/mol) [10].

HbA1c levels in children and adolescents are associated with age, duration of diabetes and gender [11], [12], [13]. The aim of the present study was to describe the distribution of HbA1c levels among patients with T1DM, and to develop age dependent percentile curves for HbA1c levels hence visual representation of the of HbA1c levels according to age in individual patients compared with a reference patient population.

## Methods

### Setting and Subjects

This is a retrospective study of HbA1c measurements of children, adolescents and young adults with T1DM, followed in Juvenile Diabetes Clinic, Maccabi Health Care Services, Israel, from January 1995 to September 2010. Data collected included gender, age at onset of diabetes, disease duration, and HbA1c levels. HbA1c levels obtained in the first six months following the diagnosis of T1DM were excluded. In addition, only data prior to a first pregnancy were included. We previously found no differences in HbA1c levels, between normal weight, overweight and obese subjects with T1DM [14], therefore data were not stratified according to weight groups.

### HbA1c measurements

HbA1c was measured at each visit either by immunological in vitro assay (Tina-quant, Boebringer Mannheim Systems) or by a fingerstick blood sample with the DCA 2000+Analyzer (Bayer Inc., Tarrytown, NY, USA) following the manufacturer's guidelines. As we previously demonstrated the DCA 2000 levels correlated well with laboratory values (r = 0.88, p<0.001) with a mean difference of DCA 2000 and laboratory HbA1c values was 0.2% (95% confidence interval −0.1–0.6%) [15].

### Statistical Analyses

Statistical analyses were performed using SAS version 9.2 (SAS institute Inc., Cary, NC, USA). Descriptive statistics are presented as number (percentages) and mean values ± standard deviation (SD).

Regression percentiles of HbA1c were calculated as a function of age using quantile regression method which was implemented with a SAS procedure QUANTREG (SAS Institute Inc, Cary, NC). (http://www.sas.com/statistics). For the quantile regression approach the polynomial model developed was: *f* (age) = *b*_0_+*b*_1_*age^½+^ *b*_2_*age^2^+*b*_3_*age^3^+… *b*_d_*age^d^ in order to estimate the mean and the conditional quantiles [16]. The highest degree *d* in the polynomial was determined by consecutive adding higher degree polynomials until the added term was no longer statistically significant. For both males and females percentiles of HbA1c were fitted by third degree polynomials. In order to calculate gender-specific percentiles the gender variable was added to this set.

Ethics Statement: In this study data were obtained retrospectively from charts, and were de-identified. Since data were analyzed anonymously, informed consent was not obtained from participants. The study and exemption from informed consent were approved by the Institutional Review Board of the Maccabi Health Care Services.

## Results

### Study Sample

During the period January 1995 to September 2010, 349 patients with T1DM were treated in the Juvenile Diabetes Center of Maccabi Health care services. Excluded from the analysis were all measurements from patients with celiac disease diagnosed by intestinal biopsy after positivity for endomysial and/or tissue transglutaminase antibody (n = 20), with eating disorders diagnosed according to the Diagnostic and Statistical Manual of Mental Disorders (DSM-IV) [17], (n = 41) and from one patient with thalassemia minor. The final study cohort comprised 287 children, adolescents and young adults with T1DM, including 155 (54%) males and 132 (46%) females. Seventeen (6%) patients had concomitant thyroid disease, 2 patients had autoimmune hepatitis and 2 patients had psoriasis. All of the patients were treated with intensive insulin treatment either pump treatment or multiple daily injections. The mean±SD age at disease onset was 9.3±5.1 years (median 9.1, range 0.7–24.5) and the mean duration of follow up was 8.7±5.6 years (median 7.1, range 0.9–27.6). The study sample comprised 4815 consecutive HbA1c measurements from 287 patients (2504 measurements for males and 2311 for females). The mean±SD number of measurements per patient was 16.8±10.6 (median 15, range 2–59).

### HbA1c percentiles according to age

Firstly, the crude 10^th^, 25^th^, 50^th^, 75^th^ and 90^th^ percentiles for HbA1c level in each age group from 2 to 30 years were calculated. Secondly, regression percentiles of HbA1c were determined by quantile regression. The modeled 10^th^, 25^th^, 50^th^, 75^th^ and 90^th^ HbA1c percentiles as a function of age for all patients, expressed in %, are shown in Table 1. The HbA1c levels for all patients, for females and for males separately in both % and mmol/mol units are provided in the supplementary files (Table S1 and Table S2).

**Table 1:** Mean ±SD HbA1c levels and the modeled 10^th^, 25^th^, 50^th^, 75^th^ and 90^th^ HbA1c percentiles as a function of age for all patients.

Age (Years)	No of measurements	HbA1c levels (%)						
		Percentiles					Mean	SD
		10	25	50	75	90		
2	34	6.9	7.3	7.7	8.2	8.7	7.8	0.7
3	65	7.2	7.7	8.2	8.7	9.7	8.3	1.0
4	89	7.0	7.3	7.9	8.5	9.1	8.0	1.0
5	112	6.6	7.1	7.8	8.6	9.3	7.9	1.1
6	144	6.5	7.0	7.8	9.0	10.0	8.1	1.4
7	164	6.8	7.1	7.8	8.7	9.5	8.0	1.4
8	205	6.5	7.1	7.8	8.6	9.2	7.9	1.0
9	215	6.3	7.1	7.9	8.6	9.3	7.9	1.1
10	236	6.6	7.2	7.9	8.6	9.4	8.0	1.2
11	270	6.7	7.3	8.1	8.9	9.6	8.2	1.3
12	246	6.7	7.5	8.1	8.9	9.6	8.2	1.2
13	245	6.7	7.4	8.1	8.8	9.6	8.1	1.2
14	276	6.6	7.3	8.0	8.9	10.1	8.2	1.4
15	287	6.5	7.3	8.0	9.0	9.8	8.1	1.3
16	315	6.4	7.1	7.9	8.8	9.6	8.0	1.4
17	259	6.3	7.1	7.8	8.7	9.5	8.0	1.4
18	245	6.5	7.1	7.8	8.5	9.3	7.9	1.2
19	220	6.5	7.0	7.5	8.3	8.9	7.7	1.1
20	189	6.0	7.0	7.5	8.3	9.0	7.6	1.2
21	154	6.2	6.8	7.5	8.0	8.6	7.5	1.2
22	142	6.2	6.8	7.4	8.3	8.8	7.5	1.1
23	124	6.2	6.7	7.2	8.0	8.6	7.3	0.9
24	118	6.1	6.7	7.3	7.8	8.2	7.2	0.8
25	103	6.3	6.6	7.1	7.7	8.4	7.2	0.8
26	105	6.3	6.8	7.1	7.7	8.1	7.2	0.9
27	81	5.7	6.2	7.1	7.7	8.0	7.0	1.0
28	73	6.3	6.7	7.1	7.6	8.2	7.1	0.8
29	50	6.1	6.6	7.2	7.6	8.1	7.1	0.7
30	49	5.7	6.5	6.9	7.4	8.1	7.0	0.8

**Figure 1:**
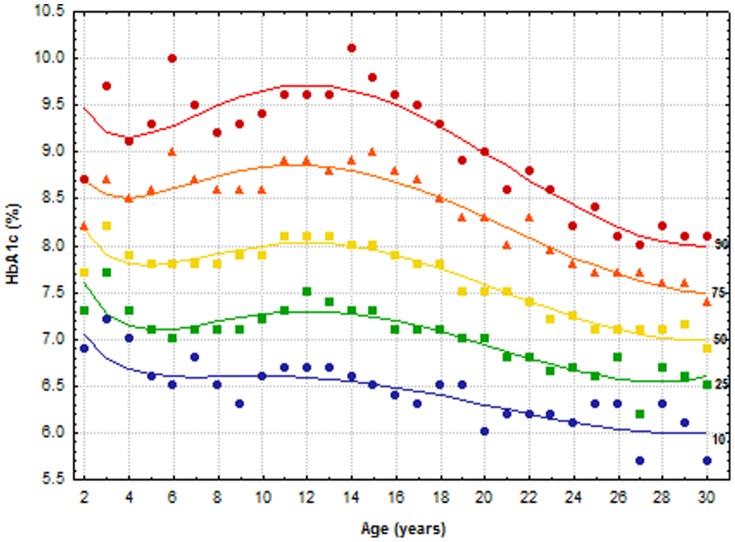
Crude percentiles of HbA1c for each age for all patients, and the modeled curves produced using quantile regression. Symbols depict the crude percentiles. To convert values for HbA1c in % to mmol/mol, subtract 2.15 and multiply by 10.929, or use the conversion calculator at www.diabetes.co.uk/hba1c-units-converter.html.

The modeled percentile curves produced using quantile regression; together with the crude percentiles of HbA1c levels for each age, for all patients are depicted in Figure 1. The figure illustrates good concordance between crude percentiles and the estimated curves. The curves show a decline in HbA1c levels from age 2 to 4 years at each percentile. Thereafter, there is a gradual increase during the prepubertal years with a peak at ages 12 to 14 years. HbA1c levels subsequently decline to the lowest values in the third decade.

### HbA1c percentiles according to gender

**Figure 2:**
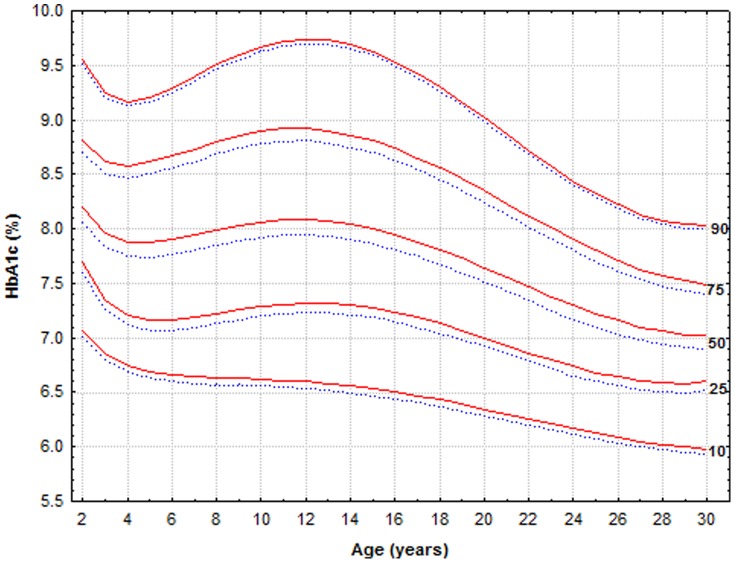
Modeled HbA1c curves for females (red) and males (blue). To convert values for HbA_1c_ in % to mmol/mol, subtract 2.15 and multiply by 10.929, or use the conversion calculator at www.diabetes.co.uk/hba1c-units-converter.html.

In order to calculate gender-specific percentiles, the gender variable was added to the regression model, including both polynomial of age and gender variables. Figure 2 depicts the HbA1c curves for females and males. The curves of females and males follow closely, with statistically significant differences between the curves; females having HbA1c levels about 0.1% higher in the 25^th^ percentile (p = 0.005), 50^th^ percentile (p = 0.0003) and 75^th^ percentile (p = 0.01). The 10^th^ and 90^th^ percentiles were similar for males and females (p = 0.29 and p = 0.61 respectively). Since a difference of 0.1% (1.1 mmol/mol) in HbA1c levels is most likely of no clinical significance we applied a single graph for males and females.

### HbA1c percentiles and the ADA age specific recommendations

**Figure 3:**
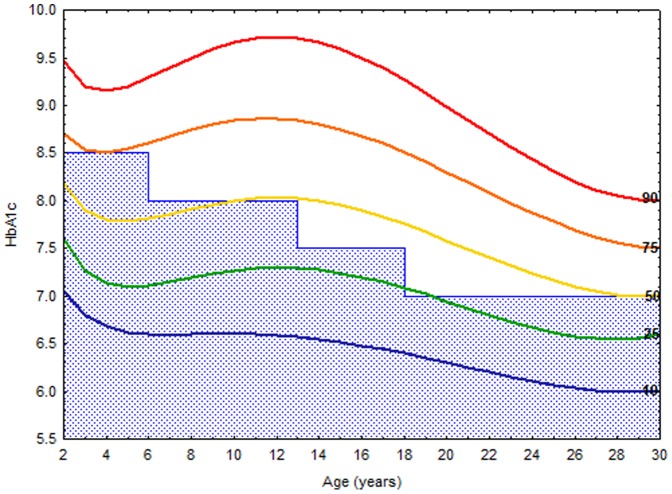
HbA1c distribution curves on the background of the ADA age specific recommendations. To co**n**vert values for HbA1c in % to mmol/mol, subtract 2.15 and multiply by 10.929, or use the conversion calculator at www.diabetes.co.uk/hba1c-units-converter.html.

Figure 3 shows the combined male and female HbA1c distribution curves on the background of the ADA age specific recommendations. Between ages 2–6 years, 75% of the measurements were within the recommended target, between 6–13 years 50% of the measurements attained the target, between 13–18 years only 25% attained the target. After age 26 about 50% of the measurements were below the recommended target level.

### HbA1c measurements plotted on reference curves

**Figure 4:**
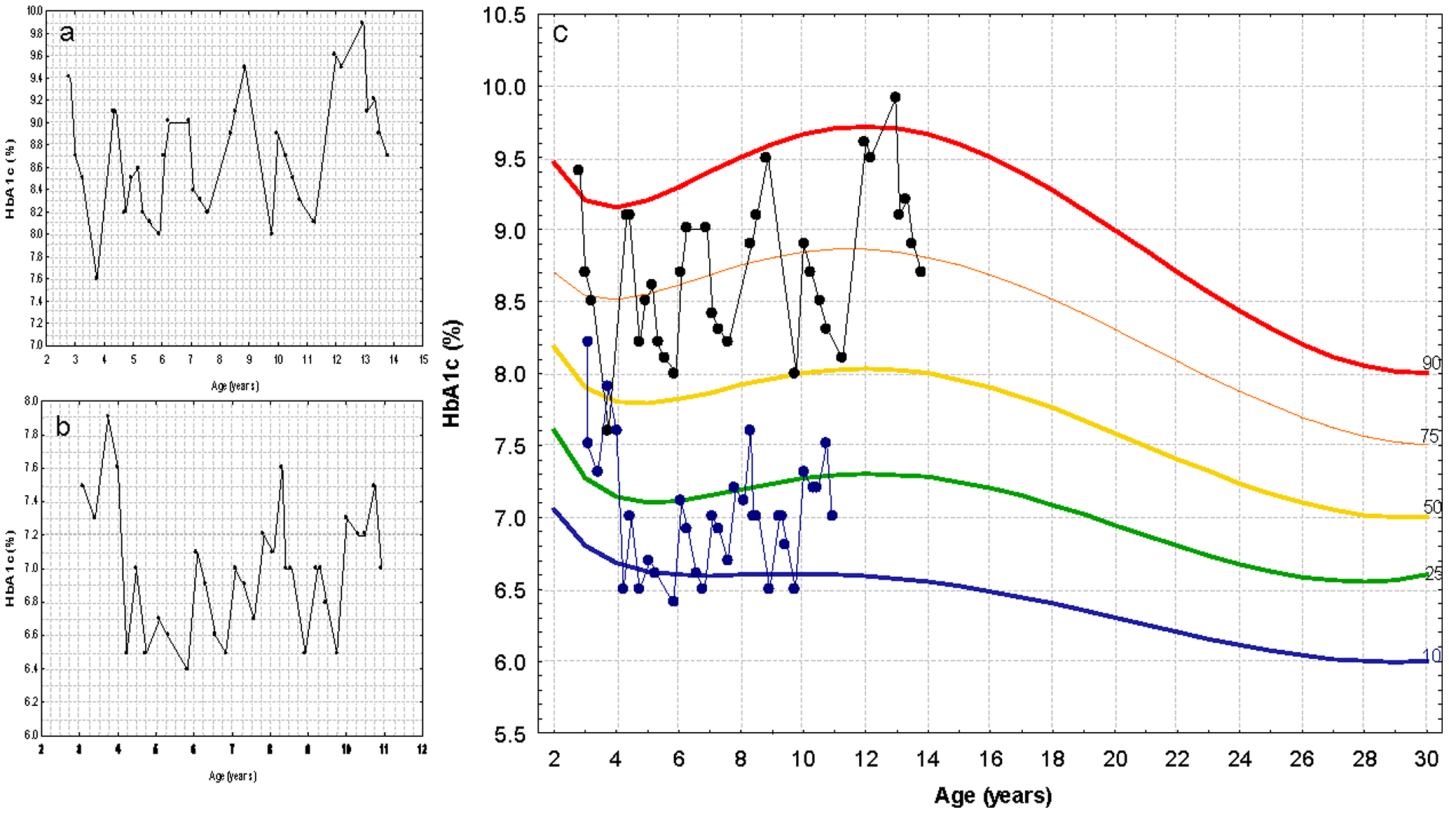
HbA1c measurements plotted on regular follow-up charts of a patient with poor metabolic control (a), good metabolic control (b), and of both patients plotted on the background of the HbA1c reference chart (c). To convert values for HbA1c in % to mmol/mol, subtract 2.15 and multiply by 10.929, or use the conversion calculator at www.diabetes.co.uk/hba1c-units-converter.html.

Figure 4 shows examples of HbA1c measurements from two individual patients as plotted both on the regular follow-up charts (a and b) and on the background of the combined HbA1c reference chart (c), emphasizing the difference between these patients. Curves for clinical and personal use are provided in Figure S1.

## Discussion

We have constructed curves of HbA1c levels using quantile regression. These curves demonstrate the distribution of HbA1c levels among T1DM patients, and can serve as a new tool to graphically illustrate an individual patient's measurements longitudinally compared with age-matched patients.

The ADA recommends a less-stringent targetfor children younger than 6 years [18], since highly variable eating and activity patterns, together with frequent illness, impose difficulties on glycemic control in this age group [19]. As ADA targets are higher, 75% of our measurements in this age group were within the recommendations. This is similar to the Type 1 Diabetes Exchange Clinic Network results where 64% of the under-6-year age group met the ADA goals [7]. At adolescence ADA recommendations are more stringent aiming to lower HbA1c levels, however our data show that during this period HbA1c levels increase to peak levels, therefore 75% of the measurement were above the ADA recommendations [18]. These findings reflect the challenge in achieving glycemic control during pubertal years. Similarly, worsening of glycemic control during puberty was observed in a longitudinal study; [20] and 60% of adolescents aged between 13 and 18 years receiving multidisciplinary care reportedly had glycemic control outside the ADA recommended target [21]. After age 18 years the distribution curves show improvement in all percentiles. Similar to our findings, mean levels of HbA1c from 117 subjects decreased from age 18 to 24 years [22]. Despite this improvement in HbA1c levels, since the target recommended HbA1c after age 18 is 7% (<53 mmol/mol), only 25% of the measurements were within the target.

Comparison between the female and male HbA1c curves demonstrated that females had higher HbA1c levels in all percentiles except in the outliers, however the difference was only 0.1%. Similar findings were observed in other large studies such as the DPV-Wiss [11], SEARCH for diabetes in youth study [5] an international multicenter study and [23] a Swedish childhood diabetes registry [24] showing that for all age groups, girls and women had higher HbA1c values compared to boys.

The distribution curves presented in this study may be helpful for several purposes. For clinicians, use of these curves may enable classification of an individual glycemic control relative to similar age group as depicted in Fig. 4. Furthermore, plotting individual's data may demonstrate response to therapeutic interventions as well as identifying an abnormal clinical course. We have recently demonstrated that adolescent girls with insulin omission eating disorder have a different pattern of HbA1c levels compared with girls with simple poor glycemic control and on this basis have proposed an algorithm for the identification of insulin omission [25].

For patients and their families, the curves may also prove useful as an educational tool. Knowledge was found to independent predictor of glycemic control among patients with T1DM, and improvement in patients' understanding of HbA1c was associated with improvement in glycemic control. [26], [27]. Strategies to convey data has been previously studied and visual explanatory cues were found to be important for people's comprehension [28]. We therefore created color codes in the percentiles such as blue and green suggest acceptable range and orange and red call for attention and alert to danger.

In assessing the results of this study a number of limitations should be considered. Firstly, the curves are based on HbA1c levels from different patients at different ages, a similarly known limitation in building longitudinal height, weight and BMI curves. In addition treatment modalities and interventions were not taken into account in our analysis. Finally, different HbA1c assays were used over the course of the study which ran from 1995 to 2010. However, analytical variation between assays or within assays over time can be expected to be equally distributed across age groups.

The strength of this study is that it provides new and simple graphs for clinicians as well as patients to use as a basis to assess and follow their glycemic control compared to other patients of the same age group and to upper ADA recommended HbA1c levels. The current graphs do however reflect our own experience in our specific population. Additional studies in different national and ethnic groups are needed, yet the novelty of the current paper is the creation of HbA1c reference curves.

In summary, we have constructed age specific reference curves for HbA1c levels for patients with T1DM to give an indication of the distribution of HbA1c values. These curves provide a comprehensive demonstration of HbA1c levels in the real world of children from infancy to young adulthood over the years following diagnosis. Using the age-based curves will give the clinician a better understanding of the likely variability of HbA1c levels in patients at specific age. In addition the curves can be used as an educational tool to graphically illustrate the patient's glycemic control both in relation to a population of patients with T1DM, as well as in relation to their own longitudinal measures.

## Supporting Information

Figure S1 **Distribution curves of HbA1C levels according to age.**
(DOCX)

Table S1 **Modeled 10^th^, 25^th^, 50^th^, 75^th^ and 90^th^ HbA1c percentiles as a function of age for all patients, for females and for males in %.**
(DOCX)

Table S2 **Modeled 10^th^, 25^th^, 50^th^, 75^th^ and 90^th^ HbA1c percentiles as a function of age for all patients, for females and for males in mmol/mol.**
(DOCX)
