# Supplementary material for: Trajectories of HbA1c Levels in Children and Youth with Type 1 Diabetes
Source: PLoS One. 2014 Oct 2;9(10):e109109. doi: 10.1371/journal.pone.0109109 (PMC4183551; doi:10.1371/journal.pone.0109109)
Supplement: Figure S1 — Distribution curves of HbA1C levels according to age. (DOCX) [file pone.0109109.s001.docx]

**Figure S1. Distribution curves of HbA1C levels according to age**

Name:
Date of Birth Date of diagnosis
