# Supplementary material for: Trajectories of HbA1c Levels in Children and Youth with Type 1 Diabetes
Source: PLoS One. 2014 Oct 2;9(10):e109109. doi: 10.1371/journal.pone.0109109 (PMC4183551; doi:10.1371/journal.pone.0109109)
Supplement: Table S1 — Modeled 10th, 25th, 50th, 75th and 90th HbA1c percentiles as a function of age for all patients, for females and for males in %. (DOCX) [file pone.0109109.s002.docx]

Table S1. Modeled 10^th^, 25^th^, 50^th^, 75^th^ and 90^th^ HbA1c percentiles as a function of age for all patients, females and males in %.

| **All** | | | | | | | **Females** | | | | | | **Males** | | | | | |
| --- | --- | --- | --- | --- | --- | --- | --- | --- | --- | --- | --- | --- | --- | --- | --- | --- | --- | --- |
| **Age**  **(years)** | **N** | **Q10** | **Q25** | **Q50** | **Q75** | **Q90** | **N** | **Q10** | **Q25** | **Q50** | **Q75** | **Q90** | **N** | **Q10** | **Q25** | **Q50** | **Q75** | **Q90** |
| 2 | 34 | 6.90 | 7.30 | 7.70 | 8.20 | 8.70 | 6 | 7.07 | 7.69 | 8.20 | 8.81 | 9.55 | 28 | 7.01 | 7.60 | 8.06 | 8.70 | 9.51 |
| 3 | 65 | 7.20 | 7.70 | 8.20 | 8.70 | 9.70 | 17 | 6.86 | 7.35 | 7.96 | 8.62 | 9.24 | 48 | 6.80 | 7.26 | 7.83 | 8.51 | 9.20 |
| 4 | 89 | 7.00 | 7.30 | 7.90 | 8.50 | 9.10 | 34 | 6.75 | 7.21 | 7.88 | 8.58 | 9.16 | 55 | 6.69 | 7.12 | 7.75 | 8.47 | 9.13 |
| 5 | 112 | 6.60 | 7.10 | 7.80 | 8.60 | 9.30 | 40 | 6.69 | 7.16 | 7.87 | 8.61 | 9.20 | 72 | 6.63 | 7.07 | 7.74 | 8.50 | 9.16 |
| 6 | 144 | 6.50 | 7.00 | 7.80 | 9.00 | 10.00 | 59 | 6.66 | 7.16 | 7.90 | 8.67 | 9.29 | 85 | 6.60 | 7.07 | 7.76 | 8.56 | 9.25 |
| 7 | 164 | 6.80 | 7.10 | 7.80 | 8.70 | 9.50 | 59 | 6.64 | 7.19 | 7.94 | 8.73 | 9.40 | 105 | 6.58 | 7.10 | 7.81 | 8.62 | 9.36 |
| 8 | 205 | 6.50 | 7.10 | 7.80 | 8.60 | 9.20 | 87 | 6.63 | 7.22 | 7.99 | 8.80 | 9.51 | 118 | 6.57 | 7.13 | 7.85 | 8.69 | 9.47 |
| 9 | 215 | 6.30 | 7.10 | 7.90 | 8.60 | 9.30 | 101 | 6.63 | 7.26 | 8.03 | 8.85 | 9.60 | 114 | 6.57 | 7.17 | 7.89 | 8.74 | 9.56 |
| 10 | 236 | 6.60 | 7.20 | 7.90 | 8.60 | 9.40 | 117 | 6.62 | 7.29 | 8.06 | 8.89 | 9.67 | 119 | 6.56 | 7.20 | 7.92 | 8.78 | 9.63 |
| 11 | 270 | 6.70 | 7.30 | 8.10 | 8.90 | 9.60 | 143 | 6.61 | 7.31 | 8.08 | 8.92 | 9.72 | 127 | 6.55 | 7.22 | 7.94 | 8.80 | 9.68 |
| 12 | 246 | 6.70 | 7.50 | 8.10 | 8.90 | 9.60 | 144 | 6.60 | 7.32 | 8.08 | 8.92 | 9.74 | 102 | 6.54 | 7.23 | 7.94 | 8.81 | 9.70 |
| 13 | 245 | 6.70 | 7.40 | 8.10 | 8.80 | 9.60 | 135 | 6.58 | 7.32 | 8.07 | 8.90 | 9.73 | 110 | 6.52 | 7.23 | 7.93 | 8.79 | 9.69 |
| 14 | 276 | 6.60 | 7.30 | 8.00 | 8.90 | 10.10 | 145 | 6.56 | 7.30 | 8.04 | 8.86 | 9.69 | 131 | 6.50 | 7.21 | 7.90 | 8.75 | 9.65 |
| 15 | 287 | 6.50 | 7.30 | 8.00 | 9.00 | 9.80 | 142 | 6.53 | 7.27 | 8.00 | 8.81 | 9.62 | 145 | 6.47 | 7.19 | 7.86 | 8.70 | 9.59 |
| 16 | 315 | 6.40 | 7.10 | 7.90 | 8.80 | 9.60 | 146 | 6.50 | 7.24 | 7.95 | 8.74 | 9.53 | 169 | 6.44 | 7.15 | 7.81 | 8.63 | 9.50 |
| 17 | 259 | 6.30 | 7.10 | 7.80 | 8.70 | 9.50 | 125 | 6.47 | 7.19 | 7.88 | 8.65 | 9.43 | 134 | 6.41 | 7.10 | 7.75 | 8.55 | 9.39 |
| 18 | 245 | 6.50 | 7.10 | 7.80 | 8.50 | 9.30 | 119 | 6.43 | 7.13 | 7.81 | 8.56 | 9.30 | 126 | 6.37 | 7.04 | 7.67 | 8.45 | 9.26 |
| 19 | 220 | 6.50 | 7.00 | 7.50 | 8.30 | 8.90 | 116 | 6.39 | 7.07 | 7.73 | 8.46 | 9.16 | 104 | 6.33 | 6.98 | 7.60 | 8.35 | 9.13 |
| 20 | 189 | 6.00 | 7.00 | 7.50 | 8.30 | 9.00 | 101 | 6.34 | 7.00 | 7.64 | 8.35 | 9.02 | 88 | 6.29 | 6.92 | 7.51 | 8.24 | 8.98 |
| 21 | 154 | 6.20 | 6.80 | 7.50 | 8.00 | 8.60 | 75 | 6.30 | 6.93 | 7.56 | 8.23 | 8.87 | 89 | 6.24 | 6.85 | 7.43 | 8.13 | 8.83 |
| 22 | 142 | 6.20 | 6.80 | 7.40 | 8.30 | 8.80 | 70 | 6.26 | 6.86 | 7.47 | 8.12 | 8.72 | 72 | 6.20 | 6.78 | 7.34 | 8.02 | 8.69 |
| 23 | 124 | 6.20 | 6.65 | 7.20 | 7.95 | 8.60 | 54 | 6.21 | 6.80 | 7.38 | 8.01 | 8.58 | 70 | 6.16 | 6.71 | 7.25 | 7.91 | 8.54 |
| 24 | 118 | 6.10 | 6.70 | 7.25 | 7.80 | 8.20 | 55 | 6.17 | 6.74 | 7.30 | 7.90 | 8.44 | 63 | 6.11 | 6.65 | 7.17 | 7.80 | 8.41 |
| 25 | 103 | 6.30 | 6.60 | 7.10 | 7.70 | 8.40 | 53 | 6.13 | 6.68 | 7.22 | 7.80 | 8.32 | 50 | 6.07 | 6.60 | 7.10 | 7.70 | 8.29 |
| 26 | 105 | 6.30 | 6.80 | 7.10 | 7.70 | 8.10 | 52 | 6.09 | 6.64 | 7.16 | 7.71 | 8.22 | 53 | 6.03 | 6.56 | 7.03 | 7.61 | 8.19 |
| 27 | 81 | 5.70 | 6.20 | 7.10 | 7.70 | 8.00 | 40 | 6.05 | 6.60 | 7.10 | 7.63 | 8.13 | 41 | 6.00 | 6.52 | 6.98 | 7.54 | 8.10 |
| 28 | 73 | 6.30 | 6.70 | 7.10 | 7.60 | 8.20 | 29 | 6.02 | 6.59 | 7.06 | 7.57 | 8.07 | 44 | 5.97 | 6.51 | 6.94 | 7.47 | 8.04 |
| 29 | 50 | 6.10 | 6.60 | 7.15 | 7.60 | 8.10 | 23 | 6.00 | 6.58 | 7.03 | 7.52 | 8.04 | 27 | 5.95 | 6.50 | 6.91 | 7.43 | 8.00 |
| 30 | 49 | 5.70 | 6.50 | 6.90 | 7.40 | 8.10 | 24 | 5.98 | 6.60 | 7.02 | 7.49 | 8.03 | 25 | 5.93 | 6.52 | 6.90 | 7.40 | 8.00 |
