# Supplementary material for: Trajectories of HbA1c Levels in Children and Youth with Type 1 Diabetes
Source: PLoS One. 2014 Oct 2;9(10):e109109. doi: 10.1371/journal.pone.0109109 (PMC4183551; doi:10.1371/journal.pone.0109109)
Supplement: Table S2 — Modeled 10th, 25th, 50th, 75th and 90th HbA1c percentiles as a function of age for all patients, for females and for males in mmol/mol. (DOCX) [file pone.0109109.s003.docx]

Table S2. Modeled 10^th^, 25^th^, 50^th^, 75^th^ and 90^th^ HbA1c percentiles as a function of age for all patients, females and males in mmol/mol.

| **All** | | | | | | | | **Females** | | | | | | | **Males** | | | | | |
| --- | --- | --- | --- | --- | --- | --- | --- | --- | --- | --- | --- | --- | --- | --- | --- | --- | --- | --- | --- | --- |
| **Age**  **(years)** | **N** | **Q10** | **Q25** | **Q50** | **Q75** | **Q90** | **N** | | **Q10** | **Q25** | **Q50** | **Q75** | **Q90** | **N** | | **Q10** | **Q25** | **Q50** | **Q75** | **Q90** |
| 2 | 34 | 52 | 54 | 61 | 66 | 72 | 6 | | 54 | 61 | 66 | 73 | 81 | 28 | | 53 | 60 | 65 | 72 | 80 |
| 3 | 65 | 55 | 59 | 66 | 72 | 83 | 17 | | 51 | 57 | 64 | 71 | 77 | 48 | | 51 | 56 | 62 | 70 | 77 |
| 4 | 89 | 53 | 54 | 63 | 69 | 76 | 34 | | 50 | 55 | 63 | 70 | 77 | 55 | | 50 | 54 | 61 | 69 | 76 |
| 5 | 112 | 49 | 52 | 62 | 70 | 78 | 40 | | 50 | 55 | 63 | 71 | 77 | 72 | | 49 | 54 | 61 | 69 | 77 |
| 6 | 144 | 48 | 51 | 62 | 75 | 86 | 59 | | 49 | 55 | 63 | 71 | 78 | 85 | | 49 | 54 | 61 | 70 | 78 |
| 7 | 164 | 51 | 52 | 62 | 72 | 80 | 59 | | 49 | 55 | 63 | 72 | 79 | 105 | | 48 | 54 | 62 | 71 | 79 |
| 8 | 205 | 48 | 52 | 62 | 70 | 77 | 87 | | 49 | 55 | 64 | 73 | 80 | 118 | | 48 | 54 | 62 | 71 | 80 |
| 9 | 215 | 45 | 52 | 63 | 70 | 78 | 101 | | 49 | 56 | 64 | 73 | 81 | 114 | | 48 | 55 | 63 | 72 | 81 |
| 10 | 236 | 49 | 53 | 63 | 70 | 79 | 117 | | 49 | 56 | 65 | 74 | 82 | 119 | | 48 | 55 | 63 | 72 | 82 |
| 11 | 270 | 50 | 54 | 65 | 74 | 81 | 143 | | 49 | 56 | 65 | 74 | 83 | 127 | | 48 | 55 | 63 | 73 | 82 |
| 12 | 246 | 50 | 56 | 65 | 74 | 81 | 144 | | 49 | 57 | 65 | 74 | 83 | 102 | | 48 | 56 | 63 | 73 | 83 |
| 13 | 245 | 50 | 55 | 65 | 73 | 81 | 135 | | 48 | 57 | 65 | 74 | 83 | 110 | | 48 | 56 | 63 | 73 | 82 |
| 14 | 276 | 49 | 54 | 64 | 74 | 87 | 145 | | 48 | 56 | 64 | 73 | 82 | 131 | | 48 | 55 | 63 | 72 | 82 |
| 15 | 287 | 48 | 54 | 64 | 75 | 84 | 142 | | 48 | 56 | 64 | 73 | 82 | 145 | | 47 | 55 | 62 | 72 | 81 |
| 16 | 315 | 46 | 52 | 63 | 73 | 81 | 146 | | 48 | 56 | 63 | 72 | 81 | 169 | | 47 | 55 | 62 | 71 | 80 |
| 17 | 259 | 45 | 52 | 62 | 72 | 80 | 125 | | 47 | 55 | 63 | 71 | 80 | 134 | | 47 | 54 | 61 | 70 | 79 |
| 18 | 245 | 48 | 52 | 62 | 69 | 78 | 119 | | 47 | 54 | 62 | 70 | 78 | 126 | | 46 | 53 | 60 | 69 | 78 |
| 19 | 220 | 48 | 51 | 58 | 67 | 74 | 116 | | 46 | 54 | 61 | 69 | 77 | 104 | | 46 | 53 | 60 | 68 | 76 |
| 20 | 189 | 42 | 51 | 58 | 67 | 75 | 101 | | 46 | 53 | 60 | 68 | 75 | 88 | | 45 | 52 | 59 | 67 | 75 |
| 21 | 154 | 44 | 49 | 58 | 64 | 70 | 75 | | 45 | 52 | 59 | 66 | 73 | 89 | | 45 | 51 | 58 | 65 | 73 |
| 22 | 142 | 44 | 49 | 57 | 67 | 73 | 70 | | 45 | 51 | 58 | 65 | 72 | 72 | | 44 | 51 | 57 | 64 | 71 |
| 23 | 124 | 44 | 47 | 55 | 63 | 70 | 54 | | 44 | 51 | 57 | 64 | 70 | 70 | | 44 | 50 | 56 | 63 | 70 |
| 24 | 118 | 43 | 48 | 56 | 62 | 66 | 55 | | 44 | 50 | 56 | 63 | 69 | 63 | | 43 | 49 | 55 | 62 | 68 |
| 25 | 103 | 45 | 47 | 54 | 61 | 68 | 53 | | 44 | 50 | 55 | 62 | 67 | 50 | | 43 | 49 | 54 | 61 | 67 |
| 26 | 105 | 45 | 49 | 54 | 61 | 65 | 52 | | 43 | 49 | 55 | 61 | 66 | 53 | | 42 | 48 | 53 | 60 | 66 |
| 27 | 81 | 39 | 42 | 54 | 61 | 64 | 40 | | 43 | 49 | 54 | 60 | 65 | 41 | | 42 | 48 | 53 | 59 | 65 |
| 28 | 73 | 45 | 48 | 54 | 60 | 66 | 29 | | 42 | 49 | 54 | 59 | 65 | 44 | | 42 | 48 | 52 | 58 | 64 |
| 29 | 50 | 43 | 47 | 55 | 60 | 65 | 23 | | 42 | 48 | 53 | 59 | 64 | 27 | | 42 | 48 | 52 | 58 | 64 |
| 30 | 49 | 39 | 46 | 52 | 57 | 65 | 24 | | 42 | 49 | 53 | 58 | 64 | 25 | | 41 | 48 | 52 | 57 | 64 |
